# High-Flow Nasal Interface Improves Oxygenation in Patients Undergoing Bronchoscopy

**DOI:** 10.1155/2012/506382

**Published:** 2012-05-20

**Authors:** Umberto Lucangelo, Fabio Giuseppe Vassallo, Emanuele Marras, Massimo Ferluga, Elena Beziza, Lucia Comuzzi, Giorgio Berlot, Walter Araujo Zin

**Affiliations:** ^1^Department of Perioperative Medicine, Intensive Care and Emergency, Cattinara Hospital, Trieste University School of Medicine, Strada di Fiume 447, 34149 Trieste, Italy; ^2^Department of Pneumology, Cattinara Hospital, 34149 Trieste, Italy; ^3^Carlos Chagas Filho Institute of Biophysics, Federal University of Rio de Janeiro, 21949-900 RJ Rio de Janeiro, Brazil

## Abstract

During bronchoscopy hypoxemia is commonly found and oxygen supply can be delivered by interfaces fed with high gas flows. Recently, the high-flow nasal cannula (HFNC) has been introduced for oxygen therapy in adults, but they have not been used so far during bronchoscopy in adults. Forty-five patients were randomly assigned to 3 groups receiving oxygen: 40 L/min through a Venturi mask (V40, *N* = 15), nasal cannula (N40, *N* = 15), and 60 L/min through a nasal cannula (N60, *N* = 15) during bronchoscopy. Gas exchange and circulatory variables were sampled before (FiO_2_ = 0.21), at the end of bronchoscopy (FiO_2_ = 0.5), and thereafter (V40, FiO_2_ = 0.35). In 8 healthy volunteers oxygen was randomly delivered according to V40, N40, and N60 settings, and airway pressure was measured. At the end of bronchoscopy, N60 presented higher PaO_2_, PaO_2_/FiO_2_, and SpO_2_ than V40 and N40 that did not differ between them. In the volunteers (N60) median airway pressure amounted to 3.6 cmH_2_O. Under a flow rate of 40 L/min both the Venturi mask and HFNC behaved similarly, but nasal cannula associated with a 60 L/min flow produced the better results, thus indicating its use in mild respiratory dysfunctions.

## 1. Introduction

During bronchoscopy hypoxemia is commonly found [1–3]. PaO_2_ usually drops approximately 20 mmHg during the procedure [1, 4], and the worst decrease occurs during bronchoalveolar lavage (BAL) [5]. Age, gender, and baseline peripheral oxygen saturation (SpO_2_) are not reliable predictive variables of hypoxemia [6] that may persist several hours after the procedure [1, 7] and increase the incidence of cardiac arrhythmia [8].

 To avoid bronchoscopy-induced hypoxemia, oxygen supply can be delivered by interfaces fed with low (6 L/min) or high gas flows. Low-flow systems supply oxygen according to the patients' respiratory pattern, which limits their use [9]. Hence, clinically high-flow interfaces are generally used, and among them the Venturi mask has been the most commonly employed device. Recently, the high-flow nasal cannula (HFNC) has been introduced for oxygen therapy in adults [10–13], as a natural extension of their use in neonates and children [14–16]. The effectiveness of both devices has been compared so far in adult human beings with acute respiratory failure in a sequential interventional study [11] and in a randomized work in patients with mild to moderate hypoxemic respiratory failure [12]. Both groups report a better performance of the HFNC device using maximum flows of 30 and 35 L/min, respectively. Additionally, to our knowledge these cannulas and heated/humidified circuits have not been used so far during bronchoscopy in adult human beings. Finally, a CPAP-like effect was reported in patients [17] and healthy volunteers [18] using a high-flow nasal cannula. Furthermore, it seems to display a flow-dependent behaviour [18].

 Thus, we aimed at determining the effects of high-flow devices on gas exchange and cardiovascular variables in patients undergoing bronchoscopy and BAL. In all instances oxygen was supplemented by a Venturi mask or by a high-flow nasal cannula. Furthermore, two gas flow rates were applied to the latter device in order to better understand its biophysical/clinical behaviour. We compared not only the different devices/flows but also the same device along the overall bronchoscopy procedure. To verify whether a CPAP could be developed by high-flow rates, healthy awake volunteers were studied.

## 2. Materials and Methods

### 2.1. Study Design

 Forty-five patients (21 females and 24 males) ranging from 37 to 83 years of age and with a body max index (BMI) ranging from 21 to 30 (Table 1) were enrolled in the study that had been approved by our institutional review board. Informed signed consent was obtained from all patients. The clinical indications for bronchoscopy were idiopathic lung consolidation (*n* = 19); lung consolidation in the course of antibiotic therapy (*n* = 10); lung consolidation in immuno compromised patients (*n* = 5); eosinophilic pneumonia (*n* = 3); collagenopathy (*n* = 2); hemoptysis (*n* = 2); Churg-Strauss syndrome, asbestosis, lymphangioleiomyomatosis, and alveolar microlithiasis (*n* = 1 in each case).

### 2.2. Methods

 All patients were selected by the Pneumology Department, Cattinara University Hospital, to undergo fibreoptic bronchoscopy and BAL fluid collection as a diagnostic tool for pulmonary disease. They were included in the study if peripheral arterial pulse oximetry (SpO_2_) was ≥90%, age ≥18 years, did not present either respiratory or cardiac failure, and were able to breathe spontaneously throughout fibreoptic bronchoscopy. Those subjects with body mass index (BMI) ≥30, tracheostomy, requiring home oxygen therapy and/or mechanical or noninvasive ventilation, nasal and/or nasopharyngeal disease, not able to clearly express themselves, and pregnancy were excluded from the study.

**Figure 1:**
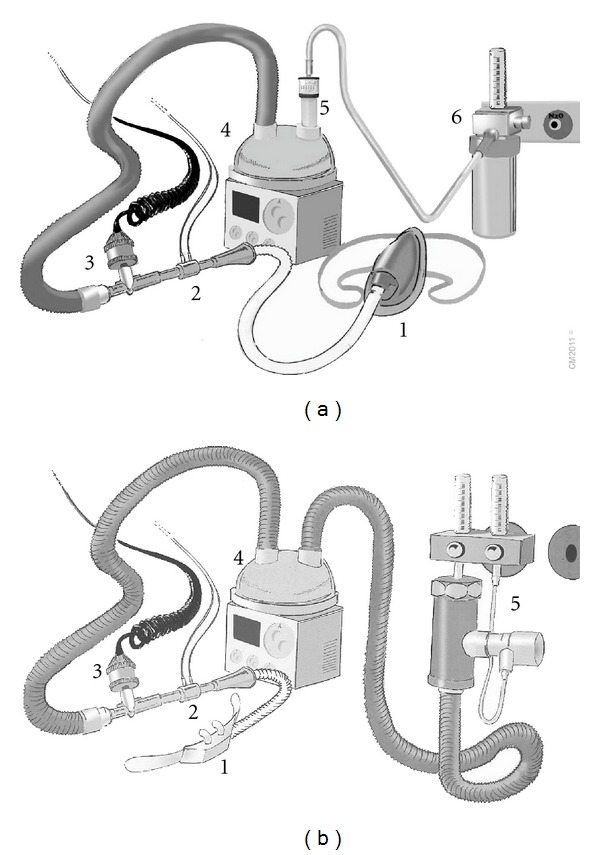
Diagram of the experimental setups. (a) Setup used for a flow rate of 40 L/min. (1) Venturi mask. (2) Pneumotachograph. (3) Oxymeter. (4) Heater/humidifier. (5) Air entrainer with the Venturi effect. (6) Wall mounted oxygen supply. (b) Setup used for delivering an airflow of 60 L/min. (1) High-flow nasal cannula. (2) Pneumotachograph. (3) Oxymeter. (4) Heater/humidifier. (5) Continuous high-flow generator with the Venturi effect.

 The patients were randomly assigned to three groups (*N* = 15 in each one) by a physician unaware of the study: groups V40 and N40 received oxygen (40 L/min, FiO_2_ = 0.5) through a Venturi mask (OS/62 K, FIAB, Vicchio, Italy) and HFNC (RT050, Fisher & Paykel, Auckland, New Zealand) during bronchoscopy, respectively (Figures 1(a) and 1(b), resp.). N60 patients also received oxygen through the aforementioned HFNC during bronchoscopy, but a higher flow rate was delivered (60 L/min, FiO_2_ = 0.5), as shown in Figure 1(b). Oxygen/air mixture in V40 group was controlled by an air entrainer with the Venturi effect (RT008, Fisher & Paykel, Auckland, New Zealand), whereas in N40 and N60 a continuous high-flow generator with Venturi effect (9293/D, Harol, San Donato, Italy) was used. In all instances the patients were in the supine position, and the administered gas mixture was humidified and warmed by a servo-controlled heated respiratory humidifier (MR730, Fisher & Paykel, Auckland, New Zealand), as depicted in Figure 1. FiO_2_ was measured on the inspiratory line by an oxymeter (5120 Oxygen Monitor, Datex-Ohmeda, Inc, Madison, WI, USA) (Figure 1). Baseline PaO_2_, PaCO_2_, pH (Rapidlab 865, Bayer, Leverkusen, Germany), SpO_2_, heart rate (HR), and non-invasive mean arterial pressure (MAP) (Dinamap, General Electrics, WI, USA) were measured during spontaneous breathing in room air (*t*_0_, Table 1). PAO_2_ was calculated by the alveolar gas equation, assuming the respiratory quotient equal to 0.8 and barometric pressure as 760 mmHg. Arterial/alveolar PO_2_ ratio (a/APO_2_) and ratio between PaO_2_ and inspiratory fraction of oxygen (PaO_2_/FiO_2_) were then arithmetically calculated. A venous catheter was indwelled to secure a line for administration of drugs and saline solution. After 5 min of oxygen (FiO_2_ = 0.5) administration, local anaesthesia (nebulised lidocaine 2%, 8–10 mL) was performed through the mouth and nostrils. A 10 min resting period was allowed to guarantee fully developed local anaesthesia. Conscious intravenous sedation was achieved by means of midazolam delivered as demanded by each patient, reaching a maximum dose of 0.1 mg/kg BW. Fibreoptic bronchoscopy (18-F, Olympus Corp, Tokyo, Japan) was immediately initiated through a dedicated mouthpiece (Pentax Europe GmbH, Hamburg, Germany). Bronchoalveolar lavage was done with 150 mL of warmed saline solution (NaCl 0.9%) and fluid was aspirated always by the same pneumologist, who had not later access to the raw data. At this point gas exchange and circulatory variables were sampled (*t*_1_). At the end of the procedure that lasted from 8 to 34 min, all patients were switched to V40 setting with a FiO_2_ = 0.35 for a resting period of 10 min. Then (*t*_2_), the last data sampling took place.

 Before discharging the patient from the bronchoscopy room, he/she was asked to describe the level of comfort during the procedure according to a scale: 1 = excellent, 2 = good, 3 = mild, and 4 = poor.

 Additionally, eight healthy volunteers ranging from 25 to 37 years of age and presenting 20 to 24 BMI (4 females and 4 males) rested in supine position and underwent local anaesthesia as aforementioned. A 35 cm long 14-F catheter (Willy Rüsch GmbH, Kernen, Germany) with two side-holes and another distal one were introduced through the nostril, its distal end reaching the hypopharynx. Its correct positioning in the pharynx was detected by gas sampling and CO_2_ monitoring (CO_2_SMO Plus 8100, Novametrix Medical System, Inc., Wallingford, CT, USA) as follows: when a normal capnographic curve resulted, the catheter was considered correctly placed; otherwise it was moved up and down until adequately positioned. The volunteers were attached to the dedicated mouthpiece (Pentax Europe GmbH, Hamburg, Germany) partially obstructed by an occluded tracheal tube (size 5, OD 6.7 mm, Rüschlit, Willy Rüsch GmbH, Kernen, Germany) that simulated the fibreoptic bronchoscope. The tracheal tube distal end was always within the mouthpiece. Oxygen (FiO_2_ = 0.28) was randomly delivered according to V40, N40, and N60 settings, and airway pressure was measured through the nasally introduced catheter by the CO_2_SMO Plus 8100 Respiratory Profile Monitor (Novametrix Medical System, Inc., Wallingford, CT, USA). A 5 min resting period was allowed between two different oxygen delivery settings. In all instances the experiment did not last more than 30 min.

### 2.3. Analysis

Statistical analysis was performed using Statistica 6.1 software (StatSoft, Vigonza, Italy). Normality was assessed by the Kolmogorov-Smirnov-Lilliefors test. Since in all instances normal distribution was not satisfied, descriptive statistics were provided using median and 1st–3rd quartiles. Mann-Whitney and the Wilcoxon tests were used to pairwise compare data among different oxygen delivery systems/flows and among diverse points along the experimental timeline, respectively. Multiple comparisons were controlled for the false discovery rate [19, 20]. In all instances, the initial significance level was set at 5%, and the adjusted *P* values are provided when significant.

A 3-sample test for equality of proportions was used to evaluate the male/female distribution. The level of comfort was assessed by permutation tests implemented in R coin package [21]. Significance level was 5%.

## 3. Results

 Patients' anthropometric and experimental data are listed in Table 1. The results will be presented firstly as function of time (*t*_0_, *t*_1_, *t*_2_) and, then, among groups (N40, N60, V40).

 At time *t*_0_ no difference among the 3 groups could be disclosed (Table 1). At the end of bronchoscopy (*t*_1_), in N60 patients a/A PO_2_, PaO_2_/FiO_2_, and SpO_2_ were larger than those in V40 and N40. In N60 PaO_2_ and PaCO_2_ were higher than those in N40 and V40, respectively. V40 and N40 did not differ between in all instances. No differences in pH, HR, and MAP values were found among the groups. Ten minutes after the end of bronchoscopy (*t*_2_), SpO_2_ between N60 and V40 was the only detected difference.

 In V40 group a/A PO_2_ and PaO_2_/FiO_2_ presented different values in all occasions. PaCO_2_ was smaller, and pH was higher in *t*_0_ than that in *t*_1_ and *t*_2_. HR was higher in *t*_1_ than in *t*_0_ and *t*_2_. In N40 group a/A PO_2_, PaO_2_/FiO_2_, and pH presented different values at all times. PaCO_2_ was smaller in t0 than in *t*_1_ and *t*_2_. HR and MAP were higher in *t*_2_ than in *t*_0_ and *t*_1_, respectively. In N60 group PaO_2_ and pH presented different values in all instances, a/A PO_2_ was higher in *t*_0_ than in *t*_1_ and *t*_2_, and PaO_2_/FiO_2_ was higher in *t*_0_ than in *t*_2_. SpO_2_ and HR were higher in *t*_1_ than in *t*_0_ and *t*_2_, and PaCO_2_ was smaller in *t*_0_ than in *t*_1_ (Table 1).

Bronchoscopy duration was similar in all groups (15, 14, and 15 min in V40, N40, and N60, resp., *P* = 0.69) as well as the amount of midazolam used (4 mg in each group, *P* = 0.95). Bronchoalveolar lavage fluid aspirated was smaller in V40 (43 mL) than in N40 (75 mL) and N60 (73 mL) that did not differ in between, (*P* = 0.0005). Gender, age and BMI did not differ among the groups (Table 1).

There was no difference among the level of comfort among V40 (level 4 = 7, level 3 = 6, and level 2 = 2), N40 (level 4 = 9, level 3 = 5, and level 2 = 1), and N60 (level 4 = 8, level 3 = 7), *P* = 0.569.

Finally, in the normal volunteers end-expiratory airway pressure amounted to 3.6 (2.4–4.0) cmH_2_O (median (1st–3rd quartiles)) using a high-flow nasal cannula and undergoing a flow of 60 L/min. In the other two experimental settings, no measurable end-expiratory pressure was detected.

## 4. Discussion

During bronchoscopy gas exchange is usually impaired owing to sedation and mismatching of the ventilation-perfusion relationship (bronchoalveolar lavage, increased airway resistance due to the presence of the fibroptic bronchoscope, and gas aspiration through the fibreoptic bronchoscope that may result in atelectasis) [22]. Hypoxemia can be treated with low- and high-flow oxygen delivery [1]. For such purpose the Venturi mask is commonly used. Recently, the high-flow nasal cannula has been introduced for oxygen therapy in adults [10–13, 23]. To our knowledge these cannulas have not been used so far during bronchoscopy in adults. Thus, we aimed at determining the effects of high-flow devices on gas exchange and cardiovascular variables in patients undergoing bronchoscopy and BAL. To verify whether a CPAP could be developed by high-flow rates, healthy awake volunteers were studied.

High-flow rates reduce the nasopharyngeal dead space, thus improving ventilation and oxygenation [11, 12, 24]. Mouth breathing may increase this phenomenon as a result of the reservoir effect produced by the mouth and nasopharynx gas volume [18]. In our patients oxygenation was further improved by a FiO_2_ equal to 50% during bronchoscopy. Furthermore, humidified and warmed high flows improve lung conductance and compliance, inhibiting bronchoconstriction and reducing the metabolic cost of O_2_ [24].

 Our 3 groups of patients presented similar demographic characteristics. Despite the statistically significant results obtained for some variables in our study, only the differences that presented clinical relevance will be discussed. In all instances, no difference in respiratory and cardiovascular measurements could be found between V40 and N40. Thus, at this flow rate both devices were equally effective. However, under N60 a/A PO_2_, SpO_2_, and PaO_2_/FiO_2_ were higher than those in V40 and N40, thus indicating a better oxygenation under these experimental conditions at the end of bronchoscopy. Indeed, in N60 a/A PO_2_ and PaO_2_/FiO_2_ did not vary significantly between the end of bronchoscopy and 10 min after bronchoscopy, whereas the values at the three experimental sampling occasions differed among them in V40 and N40. We calculated the PaO_2_/FiO_2_ and the a/A ratio because they are relatively unaffected by FiO_2_ and in particular the a/A ratio is less dependent on the patient's age [25, 26]. In this way, the absolute PaO_2_ value assumes a secondary clinical relevance.

Carbon dioxide kinetics returned to baseline values in N60 while in V40 and N40 at 10 minutes after bronchoscopy PaCO_2_ did not return to control levels. However, several studies reported different PaCO_2_ behaviours during HFNC, and thus the carbon dioxide wash-out mechanism is still not widely accepted [27] as the main physiological effect under this condition.

Possibly the development of CPAP owing to the even higher flow rate achieved with the HFNC, a smaller possibility to dilute the delivered mixture by room air, and a more constant FiO_2_ would explain these findings [27, 28]. The Venturi mask could not be tested with 60 L/min because of a technical limitation of the air entrainer itself, as stated by the manufacturer (RT008, Fisher & Paykel, Auckland, New Zealand, REF 185041357 Rev E 2009-07). Our results demonstrated that the association of HFNC and 60 L/min flow provided the better oxygenation not only during bronchoscopy but also during recovery. HFNC with smaller flow than ours also proved to be more effective than the face mask in hypoxemic respiratory failure [11, 12].

In our study we chose an oxygen delivery of 50% in order to minimize hypoxemia during bronchoscopy. After the procedure, during 10 minutes an oxygen delivery of 35% was used to evaluate the patients' recovery.

We ran a second set of experiments on healthy volunteers to verify whether a CPAP could indeed be developed by high-flow rates. This measurement was not done in the patients to avoid an undesirable extra burden during the procedure. The volunteers underwent the same three settings applied to the patients. The volunteers' median airway pressure under V40 and N40 settings was nil, but a value of 3.6 cmH_2_O was measured at end expiration under N60 conditions in volunteers with a partially obstructed mouth. Our results are in line with those previously reported. Indeed, a CPAP-like effect has been recently reported in postoperative cardiac surgery patients (2.7 cmH_2_O, 35 L/min) [17] and in normal volunteers (2.7 cmH_2_O open mouth, and 7.4 cmH_2_O closed mouth, 60 L/min) [18], and it has been demonstrated that positive nasopharyngeal pressure increases with increasing flow [18]. This CPAP could possibly contribute to the better oxygenation in our patients.

Since the level of comfort was identical in the three groups of patients, one can possibly assume that the three experimental settings were similarly supported by them. Furthermore, our patients tolerated very well the HFNC. In this line, it has been demonstrated that patients chose to continue with HFNC after having tried it [11].

In conclusion, under a flow rate of 40 L/min both the Venturi mask and the high flow nasal cannula behaved similarly, but the outcome produced by the latter associated with a flow of 60 L/min was clinically more important. Perhaps the latter association could protect to a larger extent patients with mild respiratory dysfunctions.

## Figures and Tables

**Table 1:** Anthropometric, respiratory, and cardiovascular data under facial and different nasal interfaces.

Variables	V40	N40	N60
Baseline (*t*_0_, FiO_2_ = 0.21)			

Gender (M/F)	9/6	8/7	7/8
Age (years)	68.0 (62.0–78.0)	70.0 (61.0–76.0)	64.0 (63.0–70.0)
BMI (kg/m^2^)	26.5 (22.5–29.1)	25.0 (21.4–28.0)	25.7 (21.2–28.9)
pH	7.45 (7.44–7.48)	7.47 (7.43–7.49)	7.46 (7.42–7.47)
PaCO_2_ (mmHg)	37.5 (35.0–42.1)	39.1 (37.3–41.5)	39.6 (33.4–42.5)
PaO_2_/FiO_2_	322.4 (295.6–374.3)	342.8 (295.7–371.9)	350.9 (304.3–363.8)
a/A PO_2_	0.674 (0.587–0.764)	0.723 (0.652–0.745)	0.718 (0.659–0.765)
PaO_2_ (mmHg)	67.7 (62.1–78.6)	72.0 (62.1–78.1)	73.7 (63.9–76.4)
SpO_2_ (%)	94 (93–96)	95 (91–96)	95 (93–97)
HR (bpm)	75.0 (62.0–97.0)	78.0 (72.0–85.0)	74.0 (68.0–84.0)
MAP (mmHg)	94.0 (90.0–107.0)	102.0 (92.0–112.0)	109.0 (100.0–117.0)

End of bronchoscopy (*t*_1_, FiO_2_ = 0.50)			

pH	7.41 (7.38–7.44)*	7.41 (7.38–7.44)*	7.40 (7.36–7.40)*
PaCO_2_ (mmHg)	42.7 (41.0–44.4)*	43.2 (37.9–47.6)*	43.6 (42.4–48.0)^∗,+^
PaO_2_/FiO_2_	165.0 (127.4 −199.2)*	140.6 (125.6–153.6)*	244.8 (181.6–366.8)^+,++^
a/A PO_2_	0.265 (0.207–0.326)*	0.224 (0.204 - 0.249)*	0.401 (0.295–0.604)^∗,+,++^
PaO_2_ (mmHg)	82.5 (63.7–99.6)	70.3 (62.8–76.8)	122.4 (90.8–183.4)^∗,++^
SpO_2_ (%)	94 (92–96)	92 (90–95)	98 (97–99)^∗,+,++^
HR (bpm)	90 (76–110)*	84 (80–101)	84 (70–100)*
MAP (mmHg)	108.0 (92.0–126.0)	99.0 (94.0–105.0)	103.0 (93.0–117.0)
Duration (min)	14.0 (10.0–16.0)	15.0 (12.0–16.0)	15.0 (9.0–20.0)

10 minutes after bronchoscopy (*t*_2_, FiO_2_ = 0.35)			

pH	7.42 (7.40–7.45)*	7.41 (7.39–7.44)^∗,∗∗^	7.40 (7.40–7.44)^∗,∗∗^
PaCO_2_ (mmHg)	42.2 (39.7–43.2)*	43.4 (41.0–45.7)*	40.7 (38.0–45.5)
PaO_2_/FiO_2_	248.6 (206.6–274.3)^∗,∗∗^	224.3 (206.6–249.1)^∗,∗∗^	278.8 (222.9–304.0)*
a/A PO_2_	0.441 (0.342–0.515)^∗,∗∗^	0.421 (0.352- 0.446)^∗,∗∗^	0.480 (0.389- 0.536)*
PaO_2_ (mmHg)	87.0 (72.3–101.8)	78.5 (72.3–87.2)	97.6 (78.0–106.4)^∗,∗∗^
SpO_2_ (%)	95 (92–98)	93 (91–95)	95 (95–98)^∗∗,+^
HR (bpm)	82.0 (75.0–90.0)**	80.0 (79.0–91.0)*	76.0 (64.0–89.0)**
MAP (mmHg)	91.0 (83.0–103.0)	94.0 (85.0–98.0)**	96.0 (87.0–108.0)

Values are median (1st–3rd quartiles). V40, N40: patients that received oxygen (40 L/min, FiO_2_ = 0.5) through a Venturi mask and nasal prong; respectively, N60: patients that received oxygen (60 L/min, FiO_2_ = 0.5) through a nasal high-flow interface; baseline: FiO_2_ = 0.21; end of bronchoscopy: airflow according to V40, N40, and N60, FiO_2_ = 0.5; 10 min after bronchoscopy: 15 L/min, FiO_2_ = 0.35; PaCO_2_ and PaO_2_: arterial partial pressures of CO_2_ and O_2_; respectively, BMI: body mass index; PaO_2_/FiO_2_: ratio between PaO_2_ and inspiratory fraction of O_2_; a/A PO_2_: ratio between arterial and alveolar PO_2_; SpO_2_: peripheral oxygen saturation; HR: heart rate; MAP: mean arterial pressure, duration: length of bronchoscopy. *Significantly different from *t*_0_; **significantly different from *t*_1_; ^+^significantly different from V40; ^++^significantly different from N40; significance level = 5%.
